# Traditional Chinese medicine—What are we investigating?^☆^: The case of menopause

**DOI:** 10.1016/j.ctim.2005.12.002

**Published:** 2007-03

**Authors:** Volker Scheid

**Affiliations:** School of Integrated Health, University of Westminster, 115 New Cavendish Street, Westminster, London W1W 6UW, United Kingdom

**Keywords:** Traditional Chinese medicine, Kampo, Traditional medicine, Menopause, Research design, Medical anthropology, History of medicine

## Abstract

CAM researchers commonly treat traditional medicines as unchanging systems. This article questions the validity of this approach by examining the treatment of menopausal syndrome by traditional Chinese medicine (TCM). Such treatment strategies were invented in 1964 and betray a strong influence of biomedical thinking. While they determine TCM treatment of menopausal syndrome in the West, physicians in China and Japan use many other treatment strategies from within the wider Chinese medical tradition in clinical practice. Cultural variability in the manifestation of menopausal syndrome furthermore questions the usefulness of simply importing treatment strategies from China to the West. This leads me to conclude that Chinese medicine as such can never be evaluated by means of clinical research. What we can do is use Chinese medicine as a resource for thinking about illness, and for formulating clinical interventions that may then be assessed using methods of evidence based research.

## Introduction

Eighty percent of women in the UK experience menopausal symptoms and 45% find the symptoms distressing.^1^ An increasing awareness among both health care providers and patients regarding potential adverse effects associated with hormone replacement therapy (HRT) is leading to strenuous efforts to find alternatives. This specifically includes the integration of complementary and alternative medicines (CAM) into treatment protocols tailored to women's individual needs.^1–3^

Traditional Chinese medicine (TCM) appears to be a fruitful area to which such efforts might be directed. It offers a clear and systematic approach to the treatment of menopausal syndrome that might readily be evaluated with the help of innovative research protocols being developed in the wider field of CAM research.^4,5^ These protocols are committed to establishing an evidence base for CAM. They frequently group research subjects according to TCM diagnoses, or use TCM diagnoses in their analysis. This still leaves open a host of unresolved methodological difficulties, i.e. whether or not clinical interventions should be standardized. But they do provide a platform from which such difficulties can be approached as “technical” problems by experts who share common standards rather than as “conceptual” issues that divide groups of researchers on the basis of ideological commitments.

From a sociological perspective, the development of such research protocols is indicative of a maturing discipline. Some commentators, in fact, interpret them as signs that traditional medicines everywhere are ready to be integrated into a single, global, and progressive medical system.^6^ This article seeks to challenge this analysis and the hidden ideologies and value judgments that sustain it. Using the Chinese medical treatment of menopause as an example, I argue that a collective unwillingness among practitioners and researchers to critically examine the myth of tradition that underpins the discourse on TCM and CAM – even though this critique is readily available in the anthropological and historical literature – invalidates much of the research that is currently carried out and that might be used to evaluate the TCM treatment of menopausal syndrome.

In particular, I wish to challenge the notion that CAM research is examining “Chinese medicine” or that it will ever be able to do so. As we shall see, Chinese medicine is a living tradition. Like biomedicine it offers multiple competing (if interrelated) models for thinking about illness and suffering. Physicians and patients use these models to develop treatment options that fit specific clinical contexts and, in turn, shape the understanding Chinese medicine has of itself as a tradition.^7^ Gaining familiarity with the complex histories that generate specific treatment protocols, assessing the validity of these protocols in relation to local contexts of clinical practice, and the ability to define new protocols through processes of creative development and translation thus emerge as urgent priorities in the field of Chinese medical research.

I will develop my critique in four steps. I first examine the TCM diagnosis and treatment of menopausal syndrome in the west. This demonstrates significant consensus among TCM practitioners regarding patho-physiology, diagnosis and treatment. It also shows that the further a practitioner in either China or the west moves away from access to primary sources, the more simplistic and systematic these theories and models become. In a second step I compare TCM to Chinese medicine as practiced in contemporary and late imperial China. This allows us to understand the development of treatment strategies for menopausal syndrome as contingent upon specific political factors in the Maoist China of the early 1960s. It also provides us with an understanding of the plurality of approaches to the treatment of menopausal syndrome within the Chinese medical tradition. Steps three and four contextualize TCM treatments further by exploring the cultural variability of menopausal experience and by presenting some Japanese Kampo approaches to menopausal syndrome. East Asian women experience menopause differently to women in the West, and Kampo physicians respond to these differences by utilizing traditional Chinese herbal medicine formulas in ways that is entirely different from the TCM approach. In the final section I analyze what the consequences of these observations are for CAM research on Chinese medicine and beyond.

## Traditional Chinese medicine (TCM) approaches to menopause

For the purposes of this discussion I define TCM as that interpretation of Chinese medical practice that is presented to us in contemporary Chinese medical textbooks.^8(pp6–8),9(pp107–150)^ Emerging in the late 1950s, these textbooks have been a cornerstone of state-directed efforts at modernizing Chinese medicine and integrating it into a larger health care system. In China, TCM exerts a significant influence on the practice of medicine through its dominance of educational curricula. Its influence on medical practice, however, is tempered by the continued existence of alternative resources on which clinicians can draw. These include direct access to the classical medical literature, the transmission of medical knowledge through personal master-disciple networks and lineages, and the historical plurality of the Chinese medical tradition.^8,10^ In the West, where such alternative resources are lacking, TCM is thus widely perceived to constitute mainland Chinese medicine as such, even if on the level of both teaching and practice TCM coexists with a large number of hybrid practices created in the course of the last 200 years by the complex diffusion of Chinese medicine to Europe and the West.^56–59^ This view is further enabled by a widely shared cultural discourse that perceives of traditions as homogenous, stable and unchanging. CAM research into Chinese medicine follows this trend, as TCM practices become the object of research claiming to evaluate not singular interventions for a given disorder, but “the TCM treatment” of this disorder. TCM theories about menopause, its diagnosis and treatment of menopausal syndrome, and CAM research into these practices constitute a case study that confirms these general observations.

Most Western TCM textbooks and TCM teachers^11,12^ state that Chinese medicine attributes problems of menopause to Kidney deficiency (*shen xu* ). In TCM the Kidneys (*shen* ) constitute a functional visceral system (*zang* ) to which are ascribed the regulation and sustenance of growth, maturation, and ageing. This system is sub-divided into two complementary aspects. The actualizing dynamic of Kidney yang (*shen yang* ) is imagined as the driving forces of all metabolic process. The endpoint of this process, in turn, is the production of Kidney yin (*shen yin* ), which constitutes the structive potential for the production of Kidney yang. Ageing in TCM is modelled as a diminishing vitality of this transformative cycle caused by a decline of the essence (*jing* ), which is stored in the Kidneys and underpins the functions of both Kidney yin and yang. The actual manifestations of ageing are dependent upon the relative balance between yin and yang. If yin (associated with substance, tranquillity, and moisture) declines more rapidly than yang, yang will be in relative excess producing symptoms of excess activity like hot flushes, palpitations, insomnia, forgetfulness, or dryness. If yang (associated with activity, warmth, dynamic, and metabolic function) declines more rapidly than yin, symptoms like fatigue, depression, chilliness, and oedema predominate.^13(pp140–146, 176–180)^

Because the Kidneys sustain all metabolic processes, TCM assumes that Kidney pathology eventually leads to dysfunction of other visceral systems. Hence, while Kidney deficiency is “always at the root of menopausal problems”^11^ it is often combined with secondary pathologies in practice. Those pathologies may need to be addressed in treatment but it is Kidney deficiency that constitutes the core of almost any TCM treatment protocol for menopausal syndrome. “Chinese medicine,” as one of TCM's most influential teachers in the West notes, “works by gently tonifying the Kidneys and the Kidney-Essence to help the woman in this transitional time of life.”^11^

**Figure 1:**
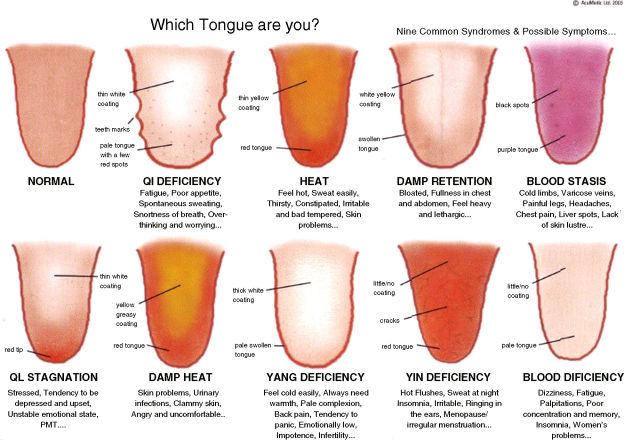
Tongue diagnosis chart employed by Acumedic Centre, London, to explain Chinese medical methods of diagnosis to potential patients.

Given the prevalence of hot flushes in the presentation of menopausal syndrome in the west, Kidney yin deficiency has come to be seen as the defining feature of menopause among western TCM practitioners. “While all patients with menopause will have Kidney yin deficiency,” writes an influential TCM teacher, “many will have other associated conditions that must be addressed.”^12^ Communicating such ideas to the wider public, they are further simplified. “In Traditional Chinese Medicine, or TCM,” the University of Maryland Medical Center informs us on its webpage, “ a woman is not generally referred to as ‘menopausal.’ Rather, a practitioner of TCM might say that she exhibits ‘[K]idney yin deficiency.”’^14^ Reversely, because hot flushes are such an emblematic and widely recognized symptom, they can also be employed to communicate to potential patients (i.e. menopausal women) how a TCM practitioner may label their condition, namely “yin deficiency” (Fig. 1).

Research investigating how TCM practitioners diagnose menopausal women with vasomotor symptoms shows that such understanding is not limited to discourse but that it consistently informs clinical practice. Zell et al.^15^ had a cohort of 23 postmenopausal women suffering from hot flushes independently successively examined by nine TCM practitioners on the same day. Practitioners diagnosed Kidney yin deficiency after 168 of 207 visits (81%). In 12 women (52%) the diagnosis Kidney yin deficiency was made by eight of nine practitioners; in 16 women (70%) by seven of nine; while in all 23 women (100%), at least five of nine practitioners made this diagnosis. There was less agreement among practitioners regarding secondary patterns of disharmony and thus about the final specific diagnosis. However, because treatment of the core pathology or “root” (*ben*) is considered the ultimate goal of treatment in TCM, such variation does not tend to fundamentally change the basic direction of treatment in clinical practice.

Suggestions for treatment of menopausal syndrome in the TCM literature, not surprisingly, are dominated by formulas and treatment protocols that tonify the Kidneys and in particular Kidney yin (Table 1). The only CAM clinical study that uses TCM disease categories as determinants of treatment^16^ likewise sorted menopausal women into Kidney yin and Kidney yang deficient treatment groups. It then employed *Liu wei di huang wan* , the most widely used TCM formula for the tonification of Kidney yin,^17(pp263–266)^ as a base formula for treatment in both groups.

My overview of the TCM and CAM literature thus allows me to draw three conclusions, which confirm the claims made in the introduction: (i) that TCM treatment of menopausal syndrome it is informed by a distinctive understanding of patho-physiology reflected in distinctive diagnostic categories; (ii) that this understanding is reflected in a broad consensus among TCM practitioners regarding core treatment protocols; and (iii) that these diagnostic categories and treatment protocols are the most likely ones to orient CAM research.

The TCM literature makes it appear that its understanding of menopause is an integral aspect of the Chinese medical tradition. In the following section, I will show that like TCM itself, the TCM understanding of menopause is a direct consequence of Chinese medical modernization.

## The invention of menopause as a medical problem in TCM

In contemporary Chinese TCM textbooks disorders related to menopause are referred to as “manifestation patterns associated with the cessation of menstruation” (*jingduan qianhou zhuzheng* ) or “symptoms and signs associated with the cessation of menstruation” (*jingduan qianhou zhenghou* ). In its visible difference from the biomedical term “menopausal syndrome” (*gengnianqi zonghezheng* ), these terms seek to emphasize a difference of both focus and origin. In terms of focus, the TCM terms draw attention to the individually specific constellation of symptoms into manifestation patterns (*zheng* ) that constitute one of the core aspects of TCM self-definition.^10^ In terms of origin, the wording “cessation of menstruation” (*jingduan* ) is a term borrowed from the classical Chinese medical literature. “Menopause” (*gengnianqi* ), on the other hand, is a direct borrowing from the Japanese term *konenki*, with which it shares the same characters, which in turn is a translation into Japanese of the German *Klimakterium* from the early 20th century.^18^ As I have shown elsewhere, such naming strategies establish legitimizing attachments to past tradition for modern innovations even as they move the Chinese medical body of process ever more closely to the biomedical body of structure.^19^

In fact, neither of the two terms used to refer to menopausal problems in TCM have any precedent in tradition. The first occurrence of “manifestation patterns associated with the cessation of menstruation” – and thus the invention of menopause as a problem for Chinese medicine – can be dated precisely to the publication of the second revised edition of *Lecture Notes for Chinese Medicine Gynaecology* (*Zhongyi fukexue jiangyi* ) in 1964. The fact that the first edition of the same text, published in 1960, did not yet include a discussion of menopause allows us to link its invention as a distinctive medical problem of concern to TCM physicians to the historical process that guided the compilation of textbooks – and thereby of TCM – in early Maoist China.^a^

Like the treatment of menopausal syndrome, textbooks emerged very late in the history of Chinese medicine. In the late 1950s, the Ministry of Health embarked on a thorough investigation of teaching materials, which culminated in a decision to edit a series of national textbooks for the teaching of Chinese medicine in 1959.^9^ The explicit goal of this policy was to systematise Chinese medicine in a manner that would make it appear more like biomedicine and thus legitimize its continued existence in the modern world. However, in the words of the historian Kim Taylor who has examined this policy in detail, it was also “a means of mass-producing future doctors of the medicine, of controlling their knowledge and practice” that “fundamentally altered the dynamics … of any form of medical innovation.”^9^

The first edition of these textbooks was written in 1959 by “teaching and research groups” (*jiaoyanzu* ) set up specifically for this purpose at various Chinese medical colleges. The *Lecture Notes for Chinese Medicine Gynaecology*^20,21^ were compiled at the Chengdu College of Chinese Medicine by a group led by Prof. Zeng Jingguang  (1918) building on teaching materials already used at the College. These included a series of handbooks published a year earlier jointly edited by Prof. Zeng and other experts from Chengdu.^22,23^ One of the most influential of these was Zhuo Qichi , a son of the locally famous Chinese medicine gynaecology expert Zhuo Yunong  who had himself studied biomedicine. Prof. Zeng had been educated at one of the new colleges of “national medicine” (*guoyi* ) during the Republican era that sought to modernise Chinese medicine while keeping its essentials in tact. In different ways, therefore, both Zeng Jingguang and Zhuo Qichi embodied the “integration of Chinese and Western medicine” actively promoted by the CCP during the late 1950s under the orders of Mao Zedong himself.^b^

Having been compiled at break-neck speed, the first edition of the new national textbooks was always intended to be provisional. It was therefore replaced after only four years by a revised and enlarged second edition. Published in 1964, these new lecture notes provided a framework for the teaching of Chinese medicine that remains influential to this date and that has been exported around the world as TCM. The revised *Lecture Notes for Chinese Medicine Gynaecology*, once more compiled in Chengdu, now discussed 44 rather than as previously 34 different disorders (*bing* ) according to the new paradigm of “pattern differentiation and treatment application” (*bianzheng shizhi* ). One of these new disorders was “manifestation patterns associated with the cessation of menstruation,” and Prof. Zeng Jingguang is today credited with its invention.

The various influences on Prof. Zeng's understanding of gynaecology are too complex for those unfamiliar with the history of Chinese medicine to outline in detail. Suffice to say that they focused on the physiology and pathology of the penetrating (*chongmai* ) and conception vessels (*renmai* ), two broad physiological functions that during the Qing dynasty had become increasingly closely associated with the uterus as the core concern of Chinese medical gynaecology. Another influence, undoubtedly, was biomedicine, assimilated during training programs run for Chinese medicine physicians in the early 1950s and at the new Chinese medicine hospitals constructed in the late 1950s.

The early 1960s, furthermore, were a particularly exciting time for those progressive physicians who embraced Mao Zedong's idea of creating a new medicine. Not only was their project supported at the highest political level but initial research suggested that it might indeed be possible to anchor Chinese medicine's body of process in the more substantial realities of physics and biochemistry. The most influential work in this respect was that of the Shanghai physician Chen Ziyin  (1928), who established that patients diagnosed as suffering from Kidney *yang* depletion (*shen yang xu* ) had consistently low urine levels of 17-hydroxicorticosteroid. This led him to propose a correlation between Kidney yang depletion and adrenal insufficiency.^24^ A connection between the Kidneys and hormonal function was thereby established in the Chinese medical imagination that persists to this day even if 40 more years of research have not fulfilled Chen's dream of matching Chinese and biomedical pathology.^c^

These observations help us to understand why menopause, which did not constitute a medical problem in the classical literature suddenly became one in 1964 and why, given the biomedical focus on menopause as hormonal decline, Chinese physicians were tempted to interpret it as a problem of deficiency. In Prof. Zeng's interpretation, however, menopause was not yet merely a problem of Kidney yin but one of “debilitated Kidney qi and deficiency and harm to the penetrating and conception vessels” (*shenqi shuairuo, chongren xusun* , ). This was to be treated by supplementing Kidney qi and regulating penetrating and conception vessels” (*bu shenqi, tiao chongren* , ).

This still left the problem of supporting the new interpretation with references to the classics, without which it could not have been considered authentic Chinese medical knowledge. Prof. Zeng did this by creating a link to chapter one of the *Inner Canon of the Yellow Lord: Basic Questions* (*Huangdi neijing su wen* ), the foundational text of the Chinese medical tradition.^d^ Its first chapter discusses the development, growth, and decline of human life in terms of seven-year cycles for females and eight-year cycles for males. Regarding women it states:

“When a woman is seven years old her Kidney qi is vigorous. The teeth are replaced and the hair grows. At fourteen, fertility is established, the conception vessel is open, the great penetrating vessel is vigorous, the menses flow, and she therefore can have children. At twenty-one, Kidney qi is fully developed. The wisdom teeth appear and [physical] growth is complete. At twenty-eight the sinews and bones are firm, the hair reaches its greatest length, and the body is luxuriant. At thirty-five, the yang brightness vessel declines, the complexion dries out, the hair begins to fall out. At forty-two, the three yang vessels decline above, the complexion is entirely dried out, the hair begins to turn white. At forty-nine, the conception vessel is depleted, the great penetrating vessel wanes, fertility is exhausted, menstruation ceases, the body has become old and she can no longer have children.”^25(p4)^

Today's gynaecology textbooks like the hugely influential *Chinese Medicine Gynaecology* (*Zhongyi fukexue* ), generally only cite the last sentence of this passage before proceeding to explain: “Around the cessation of menstruation the Kidney qi gradually declines, … hence Kidney deficiency is the root cause of this disorder.”^26(p161)^ The chief editor, Luo Yuankai  (1914–1995) from Guangdong, held a more narrow view of menopause as Kidney deficiency than Zeng Jingguang, who only was a deputy-editor, which is duly reflected in this text.^27(pp104–106)^ We thus find here a further stage in the development of the TCM understanding of menopausal syndrome. Detached from Prof. Zeng's personal focus on the penetrating and conception vessels, references to treating penetrating and conception vessels are removed and replaced by the simplified emphasis on Kidney deficiency that we also find in western TCM texts.^28(pp83–84), 29(pp126–132)^ In broader historical terms, this reduction of complexity follows the new centring of Chinese medical practice on disease “types” (*xing* ) rather than “manifestation patterns” (*zheng* ), first advanced by the above mentioned Chen Ziyin in 1973 and widely applied by modernising physicians since then.^10^

Not all Chinese medicine physicians, however, interpreted the passage from the *Inner Cannon* in the same way. For although it defines growth and development as emanating from the vigour of Kidney qi, its account of decline emphasize beyond the conception and penetrating vessels the yang channels, particularly yang brightness (*yangming* ). In clinical practice, therefore, many gynaecologists emphasize treatment of the Kidneys only in younger women, while for older women the Spleen and Stomach visceral systems, which are associated with are associated with both the yang brightness and the penetrating vessel, are considered more important. The influential Ming dynasty physician Wang Kentang  (1549–1613), whose ideas shape the practice of many contemporary gynaecologists, for instance writes:

“During their childhood, before they menstruate [and are fertile], women's [physiology] is subordinated to the lesser yin [i.e. the Kidneys]. When they menstruate [and are fertile] it is subordinated to the terminal yin [i.e. the Liver]. When menstruation [and fertility] ceases, it is subordinated to the greater yin [i.e. the Spleen].”^30(vol. 6, p1)^

The modern case record literature also provides many examples of senior physicians approaching the treatment of menopausal problems from a perspective that does not accord priority to Kidney function. The gynaecology expert Ha Litian  (1912–1989), for instance, notes that in the treatment of menopausal disorders,“… one must take the regulation of the penetrating and conception vessels as the foundation. But in order to regulate penetrating and conception vessels one must regulate the visceral systems, harmonise qi and blood.”^31^

Many other examples could be cited. My intention, however, is not to document in detail the diverse approaches to menopausal syndrome that might be found in the literature. Rather, I wish to call our intention to the complex history of invention and reinterpretation that lies beyond the surface of matter-of-fact discussions of menopause found in western TCM textbooks. What these discussions hide, too, is the plurality of approaches of treating menopausal distress in contemporary Chinese medical practice, which embody perspectives that differ from the narrow focus on Kidney deficiency cited above. If the transmission of Chinese medicine that underpins much of current CAM research continues to rely on a simplified understanding of menopausal syndrome as Kidney deficiency, then one of the reasons is the mistaken perception that textbooks prescribe actual clinical practice, as well as a lack of access to primary sources of knowledge.

Another important reason, however, could be differences between China and the west in how women actually experience the menopausal transition. For as much modern research shows, the experience of menopause as a life event everywhere is shaped not only by a universal biology but also by the distinctly local factors of culture and society.

## Menopause and culture

If, like the Oxford English Dictionary,^32^ we define menopause as “the permanent cessation of menstruation” it is a species universal, which happens sometime in the late forties or early fifties without significant variations across cultures. If, on the other hand, we use menopause to mean “the period of a woman's life when this occurs”^32^ – this, too, being part of the OED definition – subjective dimensions of experience embedded in local cultures, ideologies and histories are moved to the fore.^e^ Women in East Asian cultures like Japan,^33,34^ Singapore,^35^ Hong Kong,^36,37^ Taiwan,^38,39^ and Malaysia,^40^ for instance, do not generally associate the menopausal transition with the vasomotor symptoms (hot flushes) so emblematic of menopause in the west. Muscle and joint pains, as well as depressed moods tend to be frequently reported symptoms in these cultures but the majority of women does not consider menopause a problem requiring medical intervention. This is reflected in a relatively low uptake of HRT in countries like Hong Kong,^37,41^ Japan,^18^ or Taiwan.^42^

The reasons for such cultural difference are complex, multi-causal, and poorly understood. Besides possible genetic differences, diet and lifestyle they include perceptions of what it means for a woman to become older and how she is to manage the menopausal transition. Such perceptions are sites of contestation, shaped by diverse agencies and ideologies, and thus always in a process of transition themselves. In a sample of American menopausal women interviewed by Martin in the 1980s, older women, for instance, experienced menopause as a potentiality allowing them to face life free of the burdens of reproduction. Younger women, in contrast, appeared to have internalised the medical model of the menopausal body as being out of control and requiring support.^43^ Contemporary middle-class Asian women increasingly report symptoms that match those of Western women. Whether this is due to a shift in bodily attention stemming from a desire to be modern (i.e. Western), or whether it denotes an escape from what Bulbeck calls the “midlife silence” of Asian women – stemming from taboos concerning the discussion of sexual issues including menstruation, fear of being criticized for failure of self-discipline or expressing “menopausal madness,” and lack of information concerning how to name symptoms and lack of access to treatment – is open to question.^44^

Whatever the reasons, such differences explain why until the 1960s menopausal problems did not constitute a topic of concern to Chinese physicians. As the medical historian Charlotte Furth observes, these physicians did not even distinguish between male and female where matters of ageing were concerned:

“What moderns would understand as menopause is identified [in ancient Chinese medicine] in the same way as menarche, simply as an event in the life passage, similar in character if not in timing for males and females alike. Just as females cease to menstruate, males’ ‘semen becomes scanty’, and these changes are not seen as a ‘pathology’ but ‘part and parcel of the ungendered feebleness of old age.”^45^

Hence, what problems required treatment would have been classed under other disease categories such as “profuse uterine bleeding in old age” (*nianlao xuebeng* ), or one of the many disorders with a psychosomatic manifestation.^f^ Alternatively, they would have been treated within the family by women knowledgeable about medicine, by midwives, or by religious based non-elite medicine. These healers left few written records and their methods would be difficult in any case to integrate into the rationalistic framework of modern TCM.

Even in contemporary Chinese medicine clinics women seeking help for menopausal problems are rare. The Shanghai gynaecology expert Cai Zhuang ^46(p100)^, for instance, estimates that less than one in six (17%) of menopausal women in this most westernized of Chinese cities suffer from symptoms serious enough to require treatment. A survey of 500 menopausal women in Beijing^47^ observed that while 47% of women experienced menopausal symptoms, only 20% were taking treatment. The same survey reports that hot flushes, experienced by 23% of women, were the most commonly reported symptom. This compares with a relative high incidence of depressive symptoms among perimenopausal women in China reported by Zhao et al.^48^ The authors of this study claim that 46% of these women suffer from depressive symptoms, with 30% experiencing moderate or severe depression. The stigma attached to psychological disorder in China may explain the discrepancy between these figures.

The high incidence of depressive symptoms also, however, calls into question the entire methodology by which the TCM treatment for menopausal syndrome was constructed. Rather than beginning with the symptoms experienced by women in the clinic, this process took as its starting point a perception of ageing as decline that needed to be rectified. This contrasts not only with the classical view as documented by Furth,^45^ but also with the contemporary approach to the treatment of menopausal problems by other (i.e. non-TCM) currents of Chinese medicine. An exploration of how menopausal syndrome is treated by Kampo medicine, a Japanese medical tradition based on the use of classical Chinese herbal formulas, elucidates this difference.

## The Kampo treatment of menopausal syndrome

Kampo  (lit. “formulas from the Han dynasty”) designates a current of traditional medicine practiced in contemporary Japan that evolved out of Japanese reinterpretations of Chinese medicine. Like traditional medicine in mainland China, Kampo is multifaceted and multi-layered, consisting of diverse competing currents and styles of practice, often centred on charismatic teachers. The interrelationship between Kampo and traditional Chinese medicine in China, too, is complex and involves centuries of exchanges flowing in both directions.^50^ Tables 2 and 3 list formulas recommended for the treatment of menopausal syndrome in two different texts: one from a Chinese survey of Kampo medicine,^49^ and the second from a Kampo textbook on the treatment of menopausal disorders written for a western audience.^50^ While these texts do not represent an exhaustive listing of all Kampo styles, they suffice for the purposes of the comparisons I wish to make.^g^

The precise indication of the formulas used cannot be discussed here in detail. Columns 3 and 4 clearly demonstrate, however, how the mainstream Kampo approach differs from that of mainstream TCM. These differences can be summed up in three points: (i) The Kampo approach does not focus on the treatment of Kidney deficiency; (ii) a significant number of formulas focus on the treatment of excess rather than deficiency; and (iii) the formulas used in Kampo medicine and those recommended in TCM do not generally overlap. This implies that even though it draws on the same medical literature and thus is part of the same larger medical tradition, Kampo and TCM treat menopausal syndrome in significantly different – and sometimes diametrically opposed – ways.

Besides differences in the experience of menopause by women in China and Japan, at least two other factors may be responsible. For historical reasons explored by Lock, many Japanese gynaecologists do not share the biomedical view of menopause as an oestrogen deficiency disorder. Instead, they perceive symptoms experienced by women during perimenopause as signs of autonomic nervous system imbalances. It is likely that this perspectives also shaped the views of Kampo physicians (all of whom are trained in conventional medicine before specializing in Kampo), particularly as there exists a close affinity between the idea of autonomic nervous system imbalance and the concern for harmony that underpins all styles of Chinese medicine.^18^

A second important factor are essential differences in how TCM and Kampo practitioners match formulas to presenting complaints. In TCM treatment protocols are formulated on the basis of theories that physicians hold regarding the patho-physiology of a given condition. Kampo physicians, on the other hand, utilize a phenomenological approach that directly matches a patient's symptoms and signs to the manifestation patterns defined for specific herbal formulas. A Kampo physician thus would simply determine a woman as exhibiting a “Cinnamon Twig and Poria Pill manifestation pattern” (*Guizhi fuling tang zheng* ) with the diagnosis specifying the treatment. The ideal TCM practitioner, on the other hand, would deduce from the manifestation pattern (*zheng* ) a patho-mechanism (*bingji* ) caused by a specific disease cause (*bing yin* ). They would then devise a treatment strategy (*fa* ) to re-establish normality of physiological process and select a matching formula (*fang* ) for this purpose. In modern practice, they often simply match a disease type (*xing* ) with a formula (*fang* ).

The historical relationship between these two styles of practice is complex and need not concern us here. What is important, however, about Kampo's traditional disinterest in theory is that it is less easily recruited to biomedical models of disease. Hence, even where Kampo physicians develop new formulas for specific disorders like menopausal syndrome, they do not feel obliged to do so via the detour of Kidney deficiency. A phenomenological approach proceeding from the actual manifestations of a disorder also will be more sensitive to local variations in the experience of menopause than one positing universal processes. Hence, western TCM textbooks simply – and according to the inner logic of the system justifiably – export treatment protocols from China to the West. Kampo textbooks written for western practitioners, on the other hand, are essentially listings of formulas and their associated manifestation patterns.

## Conclusions for TCM and CAM research

Lack of space prevents me from discussing in similar detail other contemporary approaches to treating menopausal syndrome within the wider Chinese medical tradition. Table 4, which lists the formulas commonly employed in the gynaecology department of Kyung Hee University Oriental Medical Hospital in Seoul, provides just one additional perspective as well as further proof for my general thesis. Only 2 of the 10 formulas treat Kidney deficiency, while at least 5 treat excess syndromes.^52^ Many of these formulas match those used in Kampo medicine, others correspond to the Kidney tonics employed in TCM. This may reflect clinical experience. As likely, it is an expression of the historical influence that both China and Japan have exerted on Korea and its medicine.

The diversity of different approaches to menopause within the Chinese medical tradition even this very superficial account has uncovered is clearly at odds with the naturalist depiction of menopause as Kidney deficiency afforded by TCM textbooks. Such naturalism, by definition, is blind to the origins of its own vision. It claims to represent the body as it is without realising that bodies – or at least the bodies that we can become aware of as human beings – are shaped by a multitude of other factors beside biology: by local cultures and climates, dietary regimes and social mores. What menopause suggests, therefore, is that social, cultural, and physical differences among diverse populations create distinctive “local biologies,” in which disease is experienced differently and in which people are subject to different health risks.^h^

The biomedical approach to menopause, which is informed almost exclusively by attempts to understand its biology, takes scant account of such variability. Portraying itself as scientific and value-free, its definition of menopause as hormonal deficiency is nevertheless profoundly ideological. It is informed by an intrinsic gender bias that many women experience as harmful,^9^ and a perception of ageing as pathological that reflects values and not facts. When modern TCM textbooks in China thus celebrate the insertion of menopausal syndrome into the Chinese medical tradition as an important step “in the historical development of Chinese medical science,”^54^ they also, willingly or not, embrace this ideology. For this reason they only see a lack where for reasons that at least deserve exploration physicians and patients hitherto had made other choices.

Western TCM practitioners, in turn, chose to think of such modern inventions as traditional knowledge and present as an alternative to biomedicine what, in effect, is a product of its increasing domination of TCM. As long as this myth of tradition legitimised Chinese medicine among an audience sharing the same longings, such blindness may have served its purpose. Now, that the efficacy of Chinese medicine is subjected to increasingly stringent tests it may prove to be deleterious to its survival. Let us imagine for a moment that a clinical study designed to test whether a given protocol for treating Kidney yin deficiency brings relief to western women suffering from menopausal syndrome finds that this is not the case. What are the consequences? In a context where it is accepted that Kidney yin deficiency is the Chinese medicine view of menopause we draw the conclusion that Chinese medicine offers no benefits to perimenopausal women. If, on the other hand, we accept that the definition of menopause as Kidney yin deficiency is the consequence of a complex historical process into which clinical experience has constituted only one input our conclusions will be quite different. We will readily accept that the Chinese medical tradition offers many other possibilities for conceiving of menopause and that some of these alternatives may offer more fruitful avenues for treatment and research. Such research, furthermore, would then not merely be a passive process of testing given treatment protocols. Rather, it would acknowledge that a first step in developing treatment protocols is to adopt them to the contexts of specific local biologies: that protocols for women in Japan may have to look different than for those in China or the US, and that they may have to be integrated differently into different local contexts of health care delivery.

Once this is accepted, priorities of CAM research in the field of Chinese medicine are drastically changed. For what I have shown for the case of menopause is true for other conditions, too. All of TCM, and not merely some isolated parts, is a modern reconfiguration of a greater tradition. This tradition offers multiple understandings on almost anything, from the nature of disease to how a specific problem like menopause should be treated**,** even if its practitioners agree on a small number of core ideas and concepts. Rather than misconstruing Chinese medicine as a static tradition that offers specific treatments for specific conditions, CAM research must embrace the realistic and evidence-based view that it is a “tradition to think with.” That Chinese medicine as a whole can thus never be proven right or wrong, beneficial or not. What we can do is to embrace the core principles of the Chinese medical tradition in order to distil from them specific therapeutic options that then can be evaluated using the principles of evidence-based research.

The person who has most clearly elaborated these concepts and their value for contemporary practice is the twentieth century literatus physician Xie Guan  (1880–1950). His definition of the Chinese medical tradition is widely embraced by the Chinese medical community and constitutes the basis even of official government definitions of TCM.^19^ I therefore offer Xie Guan's own words as a fitting conclusion. Written almost a century ago its affinity to the notion of local biologies that modern researchers have arrived at through the examination of menopause could not be closer. How this agenda can be translated into a practical research program is a work-in-progress whose results will be published at a later date.

“To sum up [one can say that] the essentials of medicine do not lie outside the four characters principle, methods, patterns and drugs. The human body may tend towards depletion or repletion, heat or cold. In each [respective case] one decides upon warming or cooling, attacking or supplementing [methods] and administers [corresponding] prescriptions to return it to a [state of] balance. All these principles and methods can be used throughout all provinces and [also] all other countries. A person's body may be strong or weak, old or young. Disorders may be recent or long-standing, light or serious. Climates may [vary between] cold and warm, dry and moist. The environment may be hard or soft, gentle or strenuous. All these factors [can be classed] as changes of [local] situations, which [can be responded to] by collecting [different] drugs and composing [different] formulas. For in [composing] formulas and combining drugs one should follow what is appropriate to each given case and cannot [just] follow a given precedent.”^55(p62b)^

## Figures and Tables

**Table 1:** The treatment of menopause in selected TCM textbooks

Textbook title	Total number of formulas suggested	Formulas to treat Kidneys	Formulas to treat Kidney yin	Formula(s) for Kidney yin main formula(s)
*Lecture Notes for Chinese Medicine Gynaecology*^20^	3	2	1	Yes
*Chinese Medicine Gynaecology*^26^	6	6	5	Yes
*Understanding Chinese Medical Gynaecology Through Tables*^28^	2	2	1	Yes
*Encyclopedia of Treatment in Chinese Medicine Gynaecology*^29^	8	8	8	Yes
*My Sister the Moon: The Diagnosis and Treatment of Menstrual Disease by Traditional Chinese Medicine*	33	23	18	Yes
*Obstetrics and Gynaecology in Chinese Medicine*^11^	18	14	13	Yes

**Table 2:** Kampo formulas for the treatment of climacteric disorders

Name of formula	Syndrome	Pathophysiology	Excess or deficiency
Pinellia and magnolia bark decoction (*ban xia hou po tang*)	A sensation of something being stuck in the throat, attacks of palpitations, hurried breathing, uprushing of qi, chest pain, a soft abdomen, and splashing sound in the epigastric area	Phlegm	Excess
Licorice, wheat, and jujube decoction (*gan mai da zâo tang*)	Sad, cries easily, yawning, hysterical, insomnia	Restless visceral system disorder	Deficiency
Three-yellow and drain the epigastrium decoction (*san huang xie xin tang*)	No decrease of physical power, upflaring of fire, flushed red cheeks, restless mind, constipation	Yang brightness warp excess heat	Excess
Bupleurum plus dragon bone and oyster shell decoction (*chai hu jia long gû mû li tang*)	Strong constitution, restless mind, irritability and restlessness, easily angered, palpitations, insomnia, tight distension in epigastric area that is painful upon palpation	Joint disorder of the three yang warps	Excess
Augmented rambling powder (*jia wei xiao yao sân*)	Weak constitution, shoulder aches and pain, easily tired, irritability, easily angered, restless mind, heat vexation, flushed red cheeks, dizziness, insomnia	Liver blood deficiency with heat from constraint	Mixed (mainly deficiency)
Tangkuei and peony powder (*dang gui shao yao sân*)	Relatively weak constitution, cold boody, anaemic, easily tired, occasional sensations of pain in the lower abdomen, heavy head, dizziness, shoulder pain, tinnitus, palpitations, abdominal diagnosis reveals lack of strength	Blood and qi deficiency with dampness	Deficiency
Restrain the liver powder (*yi gan sân*) two-cured decoction (*er chen tang*)	Agitated, easily angered, restless sleep, soft and weak abdominal muscles, hyperactive aortic pulsation on the left side of the abdomen, resistance to pressure or even pain on palpation in left upper abdomen,	Blood deficiency with phlegm and wind-heat	Mixed (mainly deficiency)
Female spirit powder (*nu shen sân*)	Uprushing, palpitations, headache, dizziness, back feels like burnt, intense heat, sweating, depression, restlessness, long-term insomnia, constipation	Qi stagnation	Excess
Cinnamon twig and poria pill (*gui zhi fu ling wan*)	Strong constitution, red complexion, headaches, dizziness, abdominal pain, shoulder pain, palpitations, abdominal fullness, resistance to pressure or even pain on palpation of lower abdomen, irregular menstruation	Blood stasis and water accumulation	Mixed (mainly excess)
Poria, cinnamon twig, licorice, and jujube decoction (*ling gui gan zâo tang*)	Attacks of palpitations in lower abdomen, uprushing of qi from the lower abdomen to the heart and chest, accelerated breathing, dizziness, sweating from the head, palpitations	Unstable qi in lower jiao due to excess water	Mixed

From Ref. 49, p. 576.

**Table 3:** Kampo treatment of hot flushes

Name of formula	Syndrome	Pathophysiology	Excess or deficiency
Peach pit decoction to order the Qi (*tao he cheng qi tang*)	Hardness or palpable masses in lower abdomen; dark stools with normal urination; mania; heat in head; nosebleeds	Blood accumulation in the lower abdomen	Excess
Ledebouriella sage-inspired powder (*fang feng tong sheng sân*)	Signs of heat excess; tendency towards constipation; headrush syndrome; tendency toward skin problems; dark coloured urine; firm abdomen with rounded contour		Excess
Bupleurum plus dragon bone and oyster shell decoction (*chai hu jia long gû mû li tang*)	Strong constitution, restless mind, irritability and restlessness, easily angered, palpitations, insomnia, tight distension in epigastric area that is painful upon palpation	Joint disorder of the three yang warps	Excess
Coptis toxin resolving decoction (*huang lian jie du tang*)	Headrush syndrome; inflammatory conditions; irritability; restlessness; insomnia; thirst; bad breath; bitter taste; distress in upper abdomen or chest; acute bleeding; unusual warmth may be palpable in centre of chest	Heat toxin flooding the triple burner	Excess
Cinnamon twig and poria pill (*gui zhi fu ling wan*)	Strong constitution, red complexion, headaches, dizziness, abdominal pain, shoulder pain, palpitations, abdominal fullness, resistance to pressure or even pain on palpation of lower abdomen, irregular menstruation	Blood stasis and water accumulation	Mixed (mainly excess)
Female spirit powder (*nu shen sân*)	Uprushing, palpitations, headache, dizziness, back feels like burnt, intense heat, sweating, depression, restlessness, long-term insomnia, constipation	Qi stagnation	Excess
Uncaria powder (*gou teng sân*)	Headrushes; headache or heavy-headedness; dizziness; muscular tension in neck and shoulder area; ocular rubor tinnitus; thirst; nervousness; irritability; insomnia; eye fatigue or pain; poor appetite	Liver yang transforming into wind with some spleen qi deficiency and phlegm damp	Excess with some deficiency
Warm clearing beverage (*wen qing yîn*)	Dry lustreless skin; menstrual disorders; bleeding; headrush syndrome; skin problems marked by dryness, redness and itching; inflammatory conditions; anxiety; irritability; restlessness; insomnia; blood stasis signs	Blood heat or damp-heat and blood deficiency	Mixed
Poria, cinnamon twig, white atractylodes, and licorice decoction (*ling gui zhu gan tang*)	Dizziness; palpitations; shortness of breath; substernal discomfort; headaches; reduced urinary output; tinnitus; twitching; headrushes; substernal splashing sounds; mild upper abdominal rectus tension	Phlegm-mucous from spleen deficiency; water dampness with cold	Mixed
String of pearls beverage (*lian zhu yîn*)	Palpitations; dizziness (orthostatic); mild oedema; tinnitus; shortness of breath; flushing and profuse sweating; headache; soft abdominal wall with periumbilical sensation	Blood deficiency with water dampness	Mixed (mainly deficiency)
Augmented rambling powder (*jia wei xiao yao sân*)	Weak constitution, shoulder aches and pain, easily tired, irritability, easily angered, restless mind, heat vexation, flushed red cheeks, dizziness, insomnia	Liver blood deficiency with heat from constraint	Mixed (mainly deficiency)
Tangkuei and peony powder (*dang gui shao yao sân*)	Relatively weak constitution, cold boody, anaemic, easily tired, occasional sensations of pain in the lower abdomen, heavy head, dizziness, shoulder pain, tinnitus, palpitations, abdominal diagnosis reveals lack of strength	Blood and qi deficiency	Deficiency
Cinnamon twig decoction plus dragon bone, and oyster shell (*gui zhi jia long gû mû li tang*)	Fatigue; cold extremities; vasomotor symptoms; frequent urination; neurotic symptoms; periumbilical sensation; thin abdominal wall with lower or general rectus tension	Floating of empty yang with insufficiency of qi and blood	Deficiency

From Ref. 50, p. 103.

**Table 4:** Formulas for the treatment of menopausal syndrome used at Kyung Hee University Oriental Medicine Hospital, Seoul

Name of formula	Predominantly excess	Name of formula	Predominantly deficiency
Pinellia and magnolia bark decoction (*ban xia hou po tang*)	Excess (qi and phlegm)	Four substance decoction (*si wu tang*) with additions	Deficiency (blood)
Female spirit powder (*nu shen san*)	Excess (qi)	Tangkuei and peony powder (*dang gui shao yao san*)	Deficiency (blood)
Augmented rambling powder (*jia wei xiao yao san*)	Mixed (blood and qi deficiency, qi stagnation)	Rhemannia decotion with six ingredients (*liu wei di huang tang*)	Deficiency (Kidney yin)
Cinnamon twig and poria pill (*gui zhi fu ling wan*)	Excess (blood stasis with damp-cold)	Rhemannia decotion with eight ingredients (*ba wei di huang tang*)	Deficiency (Kidney yang)
Clear the heart drink with lotus seed (*qing xin lian zi yin*)	Mixed (heart fire excess with qi deficiency)		

From Ref. 52, p. 45.

## Footnotes

a The political emphasis on collectivity in early Maoist China prevented the attribution of textbooks to individuals. The editing of both texts was thus ascribed to the Chengdu College of Chinese Medicine (*Chengdu zhongyi xueyuan*).

b My account in this and following paragraphs draws on the detailed biography of Prof. Zeng.^21^

c For an overview of Chen Ziyin's research and his influence on TCM see the accounts by Ma Boying.^23^

d The dating of this text is difficult. Most of the source material was probably written in the two centuries around the turn of the millennia [BCE/CE], with its final compilation perhaps not reaching maturity until the Song dynasty.

e These issues have been discussed in detail by the anthropologist Margaret Lock.^18(pp32–38)^

f This also is the opinion of modern Chinese TCM textbooks.

g For another text that lists essentially the same formulas see Ref.^51^.

h The term “local biologies” stems from Ref.^53^.
